# Supplementary material for: Patterns of Intron Gain and Loss in Fungi
Source: PLoS Biol. 2004 Nov 30;2(12):e422. doi: 10.1371/journal.pbio.0020422 (PMC532390; doi:10.1371/journal.pbio.0020422)
Supplement: Table S1 — Also available at http://genes.mit.edu/NielsenEtAl/. (4.3 MB ZIP). [file pbio.0020422.st001.zip › NielsenEtAl/html/936.html]

AN6256.1.NCU06739.1.MG01109.1.FG06266.1


```
 CLUSTAL W (1.82) Multiple Sequence Alignments - Introns Inserted


Sequence 1: MG01109.1	155 aa
Sequence 2: FG06266.1	144 aa
Sequence 3: NCU06739.1	145 aa
Sequence 4: AN6256.1	153 aa
Alignment Length: 155 aa
Number Identitical Residues: 66 aa
Alignment Score (without introns) 3548


MG01109.1 	MTDASRWKATLYVGGLPAAATEASLHDAFIPFGEIADVKMPKNDNPKSAETHRGFAYVEY
NCU06739.1	MTDTTRWKATVHIRGLAPAVTTATIHDAFIPFGEIADISLPKNEKPDSTDPHRGFAYVEY
FG06266.1 	MSDAARWKATVHVGGLSQMATQSHVLDAFIPFGEIVEVKVPKPDAPNSTEAHRGFAYVEY
AN6256.1  	MSEKTRLKCTVYVGGLDQAVSVQTLAEAFVPFGEVVDITLPKPDLPNSNDNHRGFGYVEF
          	*:: :* *.*::: **   .:   : :**:****:.::.:** : *.* : ****.***:

MG01109.1 	EDADDAKEAMDNMDQSEFFGRIIKVHAAKPPKSANEGLGSKTAVWEQ0EGWLAKNAVSEE
NCU06739.1	EDEEDAKEAIDNMDQSELFGRVIRVSAAKIPKSAKEGLGSKTAVWEQ0EGWLAENAVSEE
FG06266.1 	EDAADAKEAIDNMDQSEFFGKVLKVSQAKAPKSADDGLGSKTAVWEK0EGWLAEHAADDE
AN6256.1  	DSPEDAKEAIDNMDGSELYGRTIKVAPAKPQKDANEGLGSKTAIWEQ0EGYLSKYAVSEE
          	:.  *****:**** **::*: ::*  **  *.*.:*******:**: **:*:: *..:*

MG01109.1 	DRLGGDQSAMATEPAPPAEDPMQGLEGLDVAGPRPE
NCU06739.1	--------DRLAATAP--DDPMQGLEGLDVAGPKPQ
FG06266.1 	--------GAVADSGP---DPMQGLEQLDVAGPKPE
AN6256.1  	DKLAAEE-SKMEDSGP-SEDPIRRLEELDVAGPKPE
          	.  ....      ..* :.**:: ** ******:*:
```
